# Double lumen endobronchial tube intubation: lessons learned from anatomy

**DOI:** 10.1186/s12871-024-02517-6

**Published:** 2024-04-19

**Authors:** Robert B. Cameron, Warwick J. Peacock, Xinlian Grace Chang, John S. Shin, Nir Hoftman

**Affiliations:** 1 Division of Thoracic Surgery, Department of Surgery, David Geffen School of Medicine at UCLA and the Division of Thoracic Surgery, Department of Surgery and Perioperative Care, West Los Angeles VA Medical Center, Los Angeles, CA USA; 2 Department of Surgery, David Geffen School of Medicine at UCLA and the Division of Thoracic Surgery, Los Angeles, USA; 3 Department of Anesthesiology, David Geffen School of Medicine at UCLA and the Department of Anesthesiology, West Los Angeles VA Medical Center, Los Angeles, CA USA; 4 Department of Anesthesiology and Perioperative Medicine, David Geffen School of Medicine, University of California, 757 Westwood Plaza, Suite 3325, Los Angeles, CA 90095 USA

**Keywords:** Airway management, Double lumen endobronchial tube, Airway anatomy

## Abstract

**Background:**

Double lumen endobronchial tubes (DLTs) are frequently used to employ single lung ventilation strategies during thoracic surgical procedures. Placement of these tubes can be challenging even for experienced clinicians. We hypothesized that airway anatomy, particularly of the glottis and proximal trachea, significantly impacts the ease or difficulty in placement of these tubes.

**Methods:**

Images from 24 randomly selected Positron Emission Tomography – Computed Tomography (PET-CT) scans were evaluated for several anatomic aspects of the upper airway, including size and angulation of the glottis and proximal tracheal using calibrated CT measurements and an online digital protractor. The anatomic issues identified were confirmed in cadaveric anatomic models.

**Results:**

Proximal tracheal diameter measurements in PET-CT scans demonstrated a mean ± standard deviation of 20.4 ± 2.5 mm in 12 males and 15.5 ± 0.98 mm in 12 females (*p* < 0.001), and both were large enough to accommodate 39 French and 37 French DLTs in males and females, respectively. Subsequent measurements of the posterior angulation of the proximal trachea revealed a mean angle of 40.8 ± 5.7 degrees with no sex differences. By combining the 24 individual posterior tracheal angles with the 16 angled distal tip measurements DLTs (mean angle 24.9 ± 2.1 degrees), we created a series of 384 patient intubation angle scenarios. This data clearly showed that DLT rotation to a full 180 degrees decreased the mean intubation angle between the DLT and the proximal trachea from a mean of 66.6 ± 5.9 to only 15.8 ± 5.9 degrees.

**Conclusions:**

Rotation of DLTs a full 180 instead of the recommended 90 degrees facilitates DLT intubations.

**Supplementary Information:**

The online version contains supplementary material available at 10.1186/s12871-024-02517-6.

## Introduction

Optimal outcomes in thoracic surgery depend on minimizing unnecessary lung trauma. One key piece of equipment used to improve outcomes during intra-thoracic operations is the double lumen endobronchial tube (DLT), which provides the ability to employ single lung ventilation strategies. Double lumen tubes are larger and more complex than single lumen endotracheal tubes and because of this necessitate specific techniques for placement. Even with significant operator experience, placement of these tubes can be challenging [1]. The difficulty of double lumen endobronchial tube placement has led to a myriad of reports describing techniques using various adjuncts such as bronchoscopy, ultrasonography, fluoroscopy, introducers/bougies, and video laryngoscopy, to facilitate placement of these devices [2–7]. Furthermore, video-equipped double lumen tubes themselves have been developed with arguable benefits [8]. Published reports have correlated tracheobronchial diameter, bronchial angulations, and aberrant anatomy to degree of difficulty of DLT placement [9–11]. Passage of the DLT through the vocal cords and subglottic airway, which may be the most challenging part of DLT insertion, has not been thoroughly studied, and can in particularly difficult circumstances lead to airway injury. In fact, such an airway injury was a major impetus for this specific study. Over a decade ago clinicians published a correspondence that described a 180 degree rotation of the DLT during glottic insertion when utilizing video laryngoscopy [12]. We adopted this technique and observed over the following decade that indeed rotating the DLT 180 degrees before passing the vocal cords and subglottis dramatically eases insertion of the DLT, during both direct and indirect video laryngoscopy. The goal of this study is to describe the anatomic and DLT factors that contribute to this clinical observation. We believe that a better understanding of the interaction between the upper airway anatomy and the configured angles of a DLT would enable better recognition of DLT insertion challenges and speed up adoption of this superior DLT placement technique.

## Methods

### Patient imaging

**Fig. 1:**
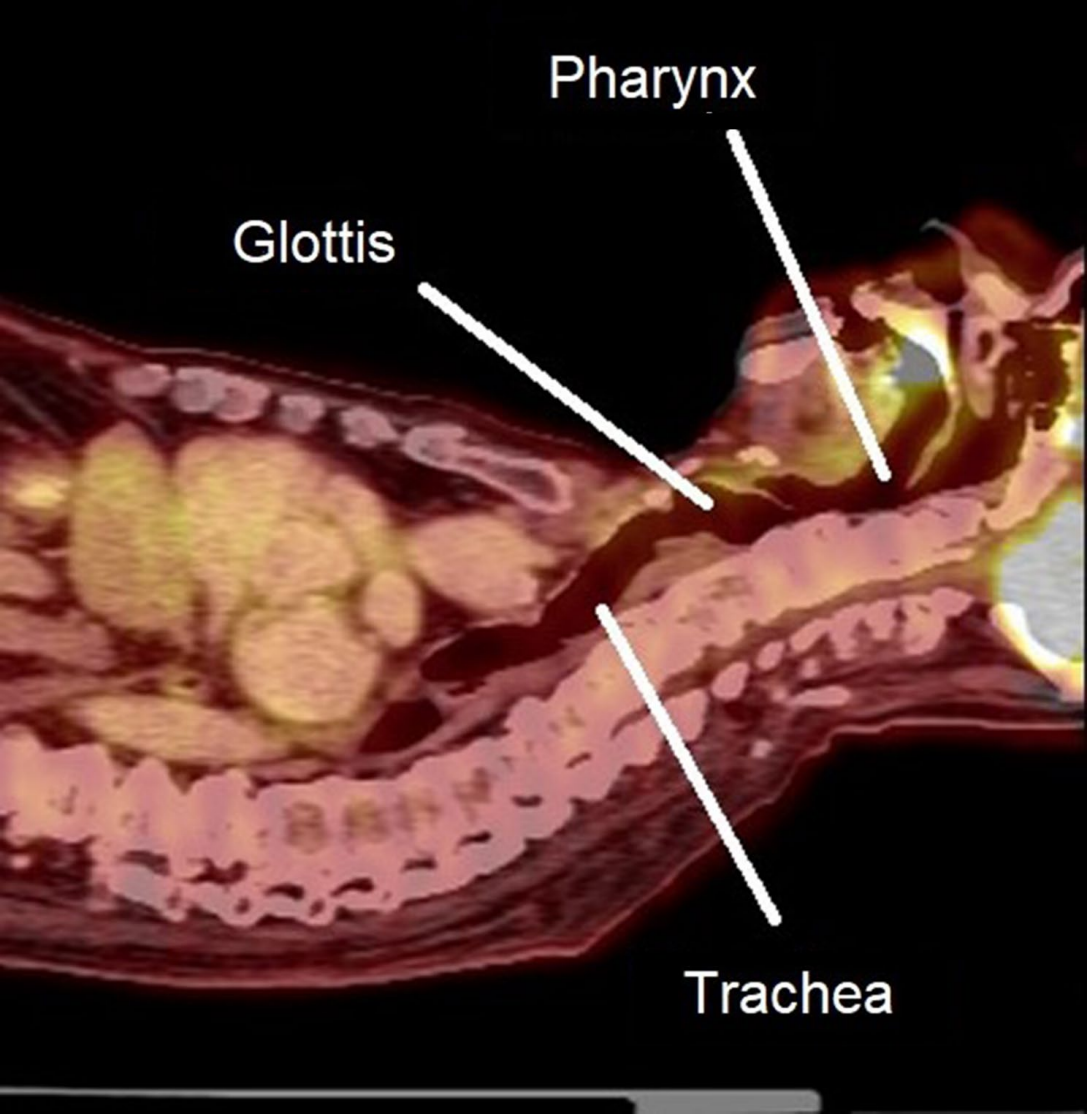
A fused sagittal image from a PET-diagnostic CT scan in a plane useful for evaluation of the airway (pharynx, glottis, and trachea)

Positron Emission Tomography-Computed Tomography (PET-CT) scans obtained in integrated PET-CT scanners from both male and female patients (*n* = 24) undergoing evaluation in the Thoracic Surgical Clinic of the David Geffen School of Medicine at UCLA were randomly selected and de-identified. Sagittal fused PET-CT images as captured in Centricity PACS RA 1000 Workstation software (GE Healthcare, Barrington, IL) which showed the best view of the entire upper airway (including the pharynx, glottis, and trachea) were chosen for further analysis (Fig. 1). Anatomic characteristics and measurements of the pharynx, glottis, and trachea were calculated.

### Calibration of fusion PET-CT image measurements

Initially, sagittal PET-CT fusion images provided measurements only in pixels and not true distance measurements. To obtain accurate distance measurements with fused sagittal images using the Centricity PACS RA 1000 Workstation software, the fusion images required calibration. Calibration was performed by measuring the length of the sternum (in cm) on the diagnostic CT portion of the scan and using this to calibrate the same measurement on the fusion images. The Centricity PACS software could then accurately calculate any measurement using the fusion images, including tracheal size measurements.

### Determination of upper airway and DLT distal tip angles

Fused sagittal images from PET-CT scans providing full body sections through the upper airway were captured using Centricity PACS RA 1000 Workstation software (GE Healthcare, Barrington, IL). These images provided optimal visualization of angles between the various parts of the upper airway, including the pharynx, glottis, and trachea. For best accuracy, angles were measured using an online digital protractor (https://www.ginifab.com/feeds/angle_measurement). Fusion images as well as other images used in the study, such as images of double lumen endobronchial tubes, were uploaded onto the digital site and the digital protractor was manipulated to the correct location for the measurement. Lines defining both parts of the angles were then created and the protractor provided accurate measurements of all angles to the nearest whole degree.

### Anatomic correlation

Donated cadavers from the Surgical Anatomy Laboratory of the David Geffen School of Medicine at UCLA underwent evaluation to confirm the anatomic relationships of the pharynx, glottis, and trachea as measured with PET-CT scans. Further, cadaveric models were used to observe a double lumen tube as it passes through the glottis and into the upper trachea. To achieve similar visualization to that provided by the PET-CT scans, the left side of the airway was surgically exposed and a portion of the pharyngeal, laryngeal, and tracheal wall were removed in order to visualize the passage of both single lumen endotracheal and double lumen endobronchial tubes. This exposure did not destabilize the anatomic structures of the airway in any detectable manner.

### Institutional review board

This research activity was approved as minimal risk by the UCLA Institutional Review Board and determined not to require patient consent (IRB#22–000712). Informed consent for the use of video and still images was obtained from the patient seen in the supplemental video.

### Statistics

Continuous variables were confirmed to be normally distributed by Excel functions NORM.DIST and NORM.S.DIST and then analyzed for significance using a student’s T test.

## Results

### Sternal calibration measurements

**Fig. 2:**
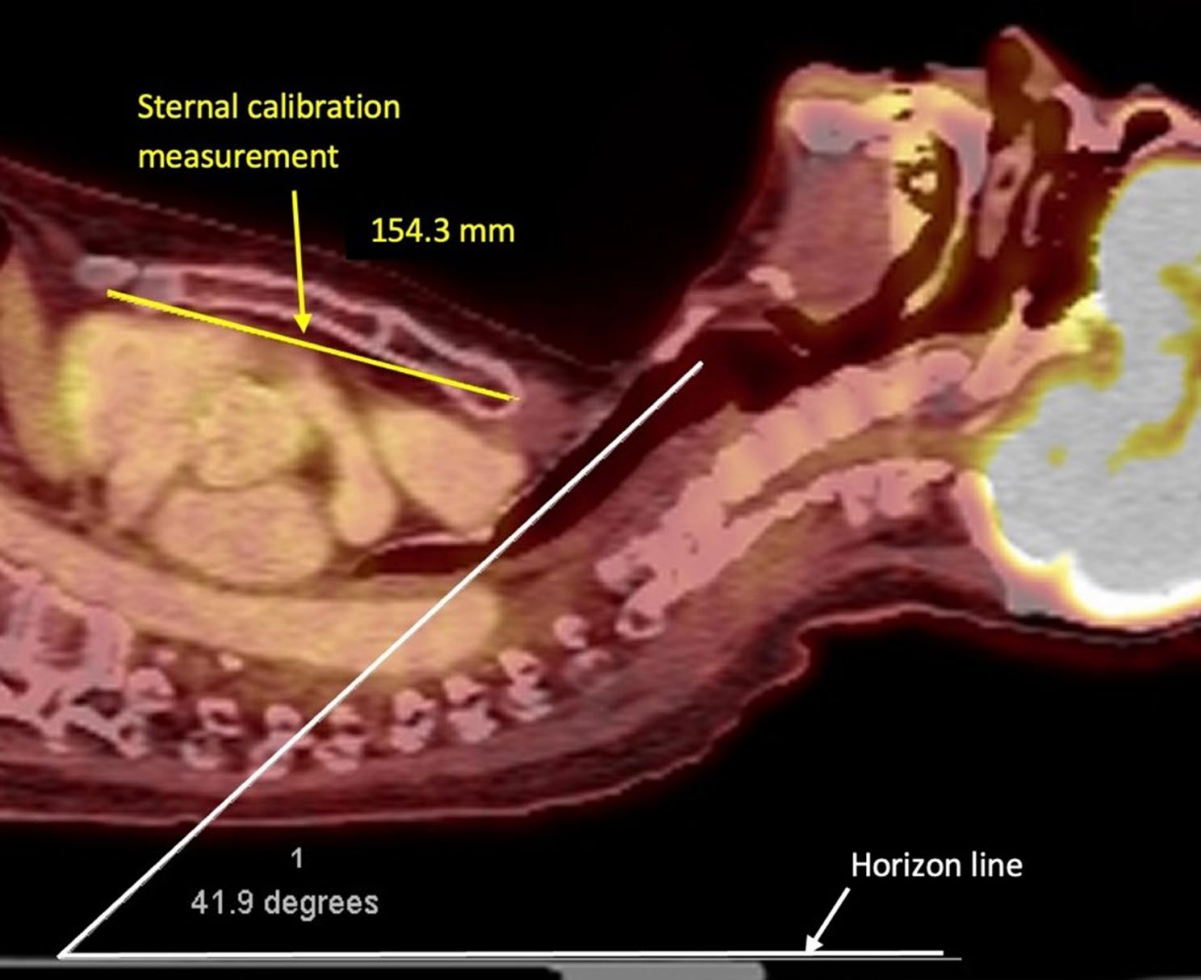
Example of sternal calibration (yellow line) and glottic/tracheal angulation (white lines)

The length of the manubrium and body of the sternum together was utilized to calibrate distance measurements in nuclear medicine fusion images, which prior to calibration were expressed only in pixels (Fig. 2). The overall mean sternal length in 24 randomly selected patients measured by a blinded investigator was 150.9 ± 17.9 mm range 119.1-185.3 mm). The mean sternal length was 162.3 ± 10.1 mm (range 144.2–180.2 mm) in 12 males and 139.4 ± 16.8 mm (range 119.1–185.3 mm) in 12 females (*p* = 0.001).

### Airway size

**Fig. 3:**
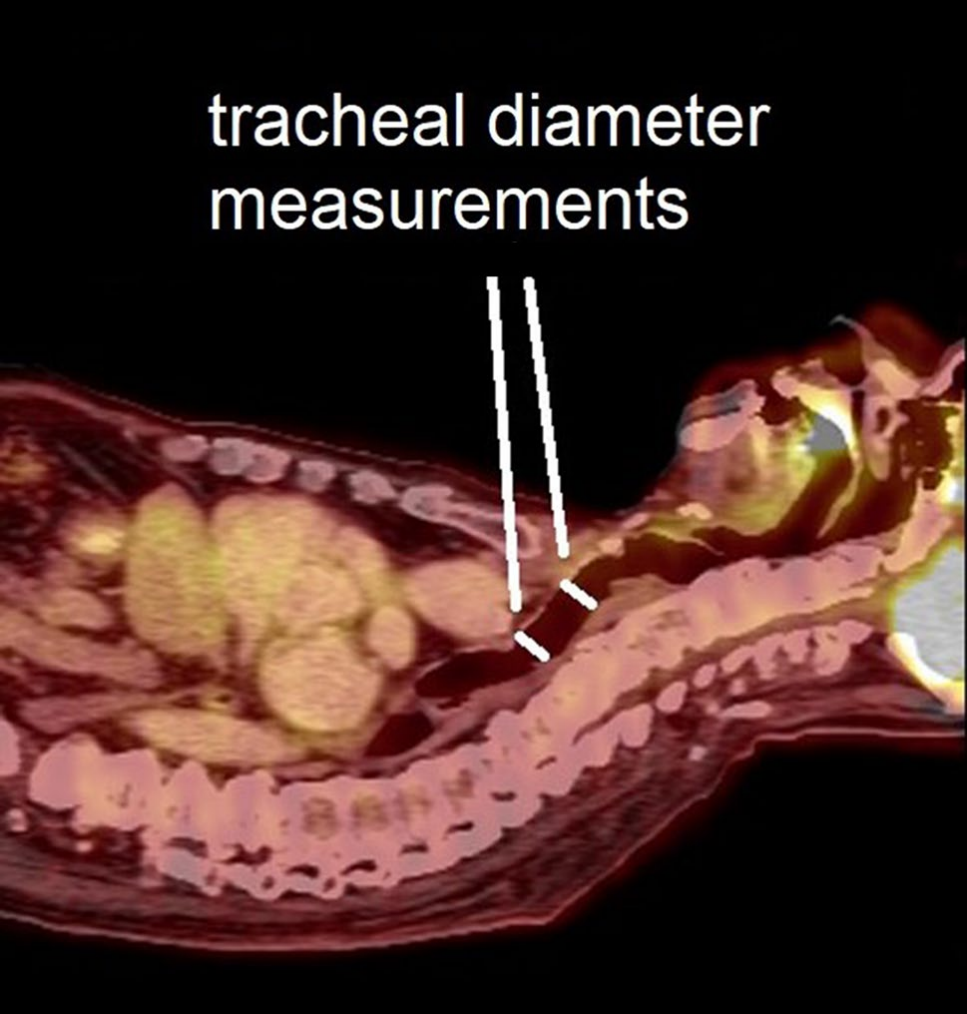
Tracheal diameter measurements performed in the proximal tracheal angulation

In order to exclude the possibility that some patients’ airways were too small for large DLTs (39 French for males and 37 French for females), we measured two independent diameters of 12 male and 12 female patients’ tracheas (Fig. 3). The overall mean ± standard deviations for all measurements was 18.0 ± 3.1 mm (range 13.8–24.5 mm); male tracheas measured 20.4 ± 2.5 mm (range 15.7–24.7 mm) and female tracheas measured 15.5 ± 0.98 mm (range 13.8–18.5 mm; *p* < 0.001). The minimum tracheal diameters for males and females were 15.7 mm and 13.8 mm, respectively. These measurements correspond to 47 F and 41 F, demonstrating that even the smallest measured male and female tracheas can comfortably accommodate their respective 39 French and 37 French double lumen tubes. Furthermore, the posterior tracheal membrane is flexible and accommodating, likely allowing for even larger tubes than this calculation would suggest. Thus, this data strongly suggests that the sheer size (diameter) of double lumen endobronchial tubes is not the major factor in the vast majority of DLT intubation difficulties.

### Airway angulation

With simple inspection of the upper airway both with PET-CT sagittal fusion images and cadaveric dissection, the angle between the pharynx and glottis was noted to be negligible and essentially equal to that of the horizon. However, the trachea turned posteriorly relative to the orientation of the glottis resulting in a significant angulation issue (Fig. 2). The overall posterior angle of the trachea as compared to the pharynx/glottis (horizon) was 40.8 ± 5.7 degrees (range 30.4–50.1 degrees) with no difference between males 40.7 ± 5.8 degrees (range 31.3–47.4 degrees) and females 40.8 ± 6.0 degrees (range 30.4–50.1 degrees).

### Double lumen endotracheal tube angulation

**Fig. 4:**
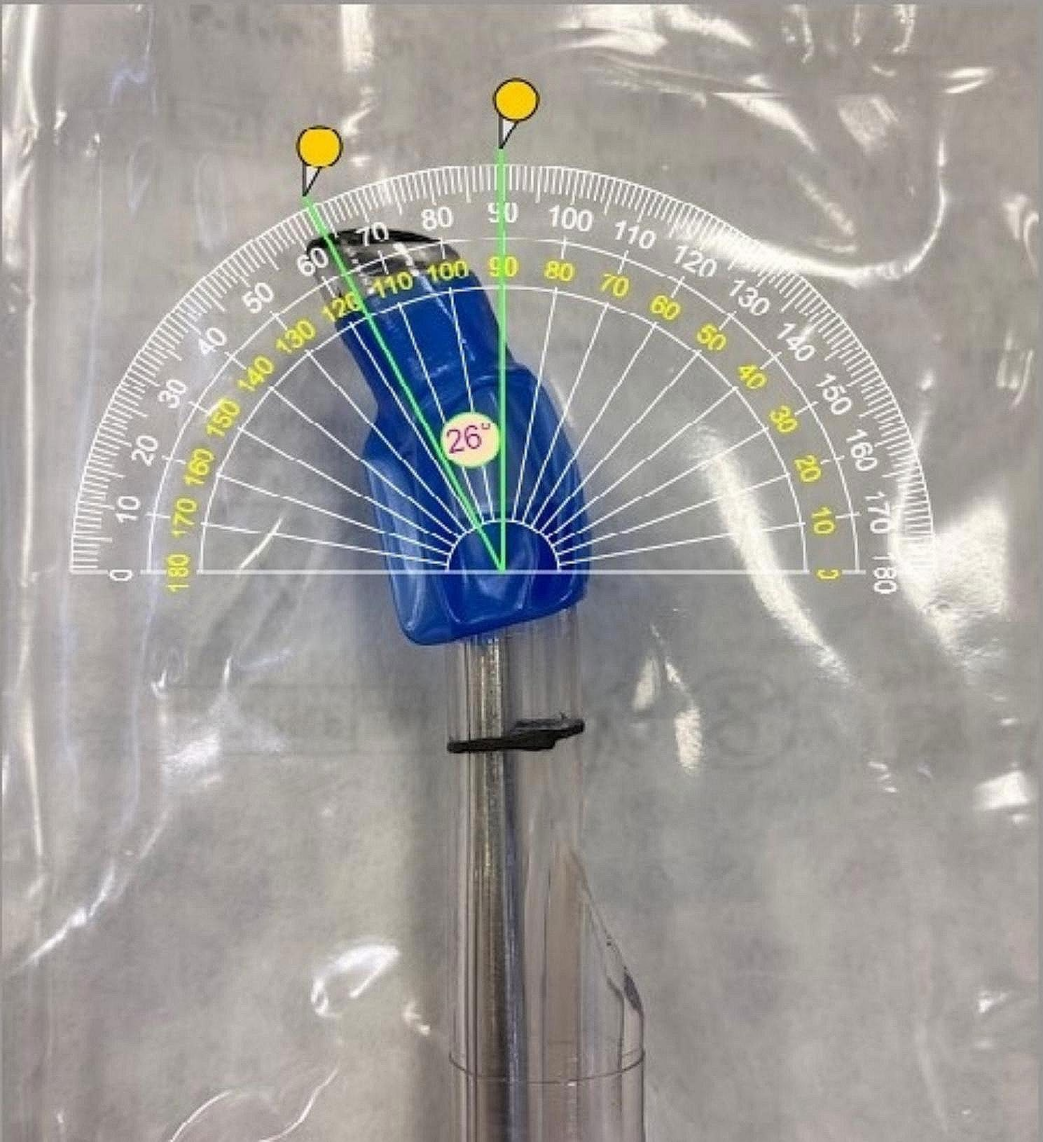
Digital protractor measurement of a DLT distal tip angle

Sixteen standard Robert Shaw type double lumen endobronchial tubes (Medtronic, Minneapolis, MN) were inspected before any manipulation, and the angulation of the distal tip measured. The tubes were photographed and the angles were measured using the online digital protractor (Fig. 4). The mean tip angle was 24.9 ± 2.1 degrees (range 21–28 degrees).

### Combinations of tracheal intubation angles

**Fig. 5:**
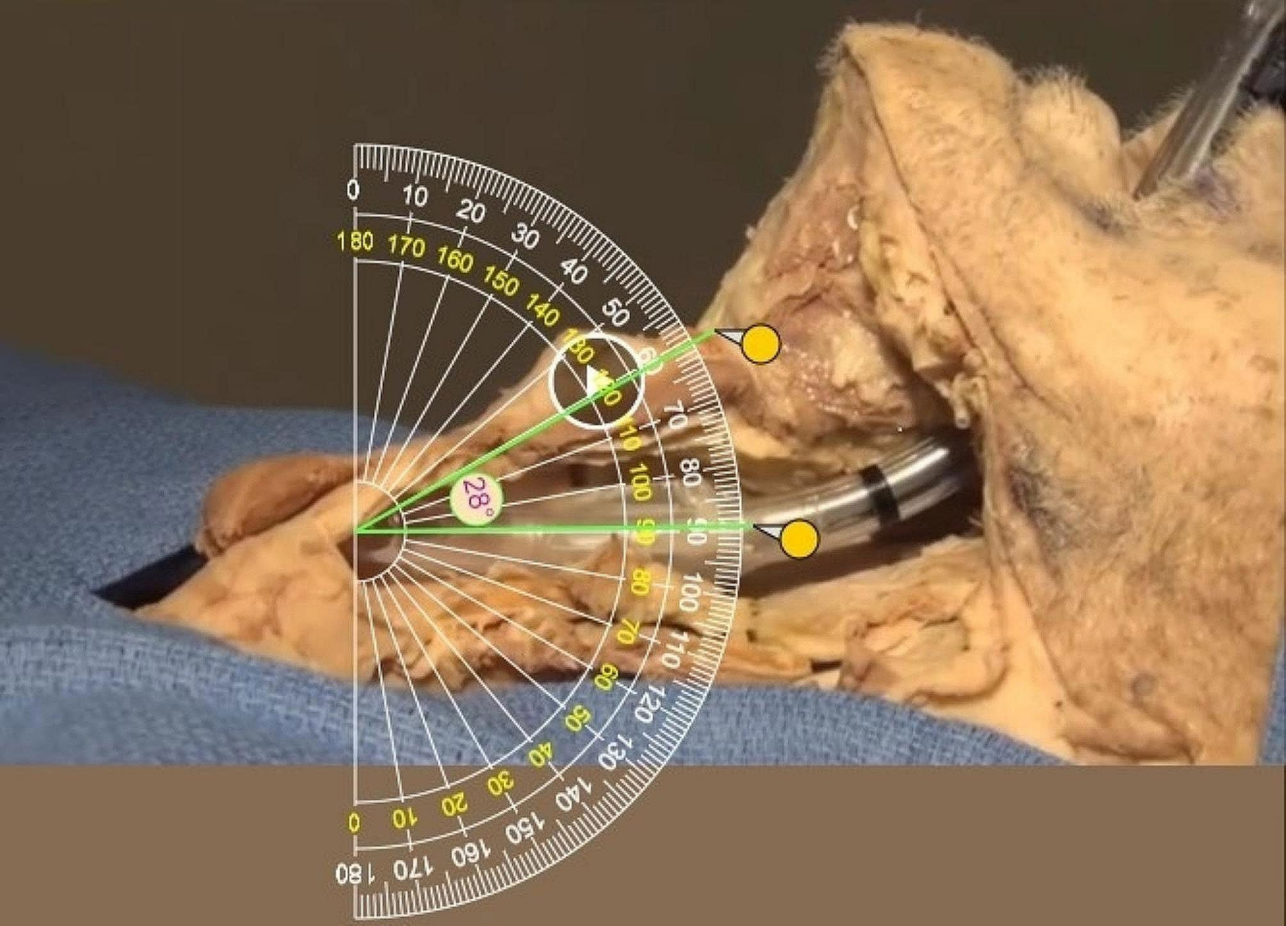
Digital protractor measurement of the proximal tracheal angulation during single lumen endotracheal intubation

During standard intubations with double lumen endobronchial tubes, the distal tip of the device is placed through the vocal cords with the tube angle oriented anteriorly. This is opposite to the posterior angulation (relative to the glottis) of the proximal trachea. We combined individual DLT tip angulation measurements (*n* = 16) with individual patient airway angulation measurements (*n* = 24) in all combinations to calculate a series of patient intubation angle scenarios (*n* = 384). The addition of these two angles together produced a steep mean angle between the axis of the double lumen tube and the axis of the glottis/proximal trachea of 66.6 ± 5.9 degrees (range 51.4–78.1 degrees). On the contrary, if the double lumen endotracheal tube is rotated 180 degrees to face posteriorly in the same direction of the trachea, the mean tube-airway angle difference decreases to a mean of only 15.8 ± 5.9 degrees (range 2.4–29.1 degrees). This compares favorably to the mean angle of 28 degrees as measured during a standard single lumen endotracheal tube intubation (Fig. 5).

## Discussion

Placement of typical Robert Shaw double lumen endobronchial tubes is frequently perceived as significantly more difficult than single lumen endotracheal intubation. Typically, this difficulty is ascribed to the large size of the tubes, but our study clearly suggests that much of the difficulty originates from airway and endobronchial tube angulation mismatch. The normal angle (approximately 28 degrees) and beveling of a single lumen endotracheal tube generally produces an angle that is not acute enough to prevent the tube from entering the posteriorly angled trachea. The standard single lumen tube is also more flexible and can navigate these angles without much difficulty. Double lumen tubes are bulkier and stiffer, making them less likely to bend around acute angles. Further, the DLT tip with its additional 24.9 ± 2.1 degrees of angulation makes a significantly more acute angle of 66.6 ± 5.9 degrees with the anterior tracheal wall. These two factors together inhibit smooth passage of the DLT into the distal trachea, often leading the device to appear stuck at or just past the glottic inlet. Finally, the corrugated anterior tracheal wall with cartilaginous rings can catch the tip of the highly angled endobronchial tube making passage even more challenging.

**Fig. 6:**
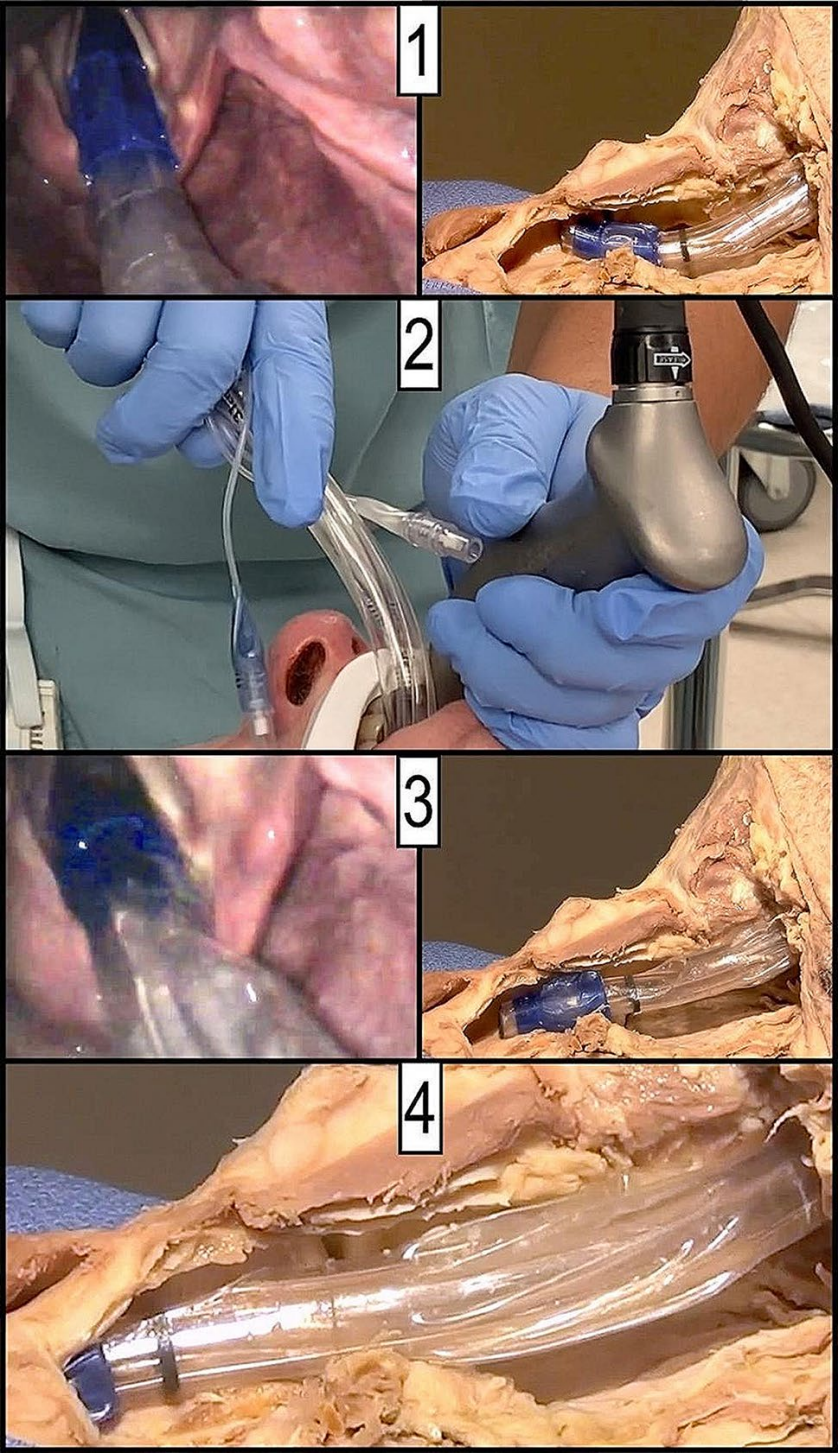
Suggested steps in double lumen endotracheal tube intubation. (1) Advance the tip of the double lumen tube facing anteriorly into the glottic opening. (2) Remove stylet and straighten any bend in the tube as much as possible avoiding any forward motion. (3) Rotate the tip of the tube a full 180 degrees to face posteriorly again avoiding forward motion. (4) Carefully and with little force advance the tube until the tracheal balloon is entirely within the airway, then rotate tip back 90 degrees to the designated side (L vs. R) and advance to the desired bronchus. Confirm final position with flexible bronchoscopy

This information, coupled with many years of positive clinical experience, leads to a proposal for a modified technique for double lumen endobronchial tube intubation involving the rotation (before attempting any advancement) of the device 180 degrees to face directly posteriorly in the same direction of the trachea with a manageable intubation angle of 15.8 ± 5.9 degrees. Extending the neck and dropping the angle of the DLT shaft to relatively match the angle of the oropharyngeal path enables smooth insertion as the DLT is rotated 180 degrees. We find this technique to improve DLT intubation with both the direct and indirect video laryngoscopy techniques, and for both left and right-sided DLTs. The maneuver (as shown in the supplemental video) facilitates insertion of the tube into the trachea and bronchus by aligning the device and airway angles before the DLT is advanced distally. Rotation of only 90 degrees after passing the tube through the glottis, as has been classically taught in the discipline [13], is not enough to disengage the endotracheal tube from the anterior cartilaginous airway. Forcefully advancing the tube without first disengaging it from the anterior tracheal wall (especially if stylet is not removed) can lead to airway damage and even catastrophic airway injury (as also shown in the supplemental video). Once the DLT passes the subglottis the shaft can be rotated 90 degrees so that its tip is facing the desired mainstem bronchus (left side for L DLT and right side for R DLT). The modified double lumen endobronchial tube intubation procedure is demonstrated in its entirety in the supplemental video and summarized in Fig. 6. Over many years using this technique and teaching it to trainees, we have found the most useful tool to be watching the demonstrated recording (supplemental video). This demonstration allows the clinician to understand visually why the 180 degree rotation is helpful.

This study does have limitations. First, the numbers of patients studied is small, and greater numbers of patients might reveal additional different anatomic variation and issues not detected by our study. Despite this, we were encouraged by the general uniformity of our data measurements and believe that additional patient numbers would not substantially change our findings. Second, we did not investigate patients with prior head and neck surgery, radiation therapy, or other diseases which could markedly alter the anatomic conditions of the upper airway. Third, we did not evaluate multiple double lumen tubes from different manufacturers, and we did not independently assess various insertion techniques, such as bougie- and bronchoscopic-assisted techniques. Fourth, we did not compare this technique to the standard insertion technique directly in this study. Since this is primarily an anatomical study, we did not collect any data on specific clinical outcomes from our proposed modified technique. The goal of this study was to determine the scientific mechanisms behind this well-established but poorly utilized insertion technique rather than compare its efficacy to the standard technique, which we believe is inferior based on extensive clinical observation. Even with these potential shortcomings, we believe our modified technique rotating the double lumen tube 180 degrees, rather than 90 as is often recommended, will significantly improve the ease and safety of double lumen endobronchial tube placement.

### Electronic supplementary material

Below is the link to the electronic supplementary material.

Supplementary Material 1

Supplementary Material 2

## Data Availability

The datasets used and analyzed during the current study are available from the corresponding author on reasonable request.

## Glossary of terms

DLT: Double Lumen Endobronchial Tube
PET-CT: Positron Emission Tomography – Computed Tomography
