# Supplementary material for: Double lumen endobronchial tube intubation: lessons learned from anatomy
Source: BMC Anesthesiol. 2024 Apr 19;24:150. doi: 10.1186/s12871-024-02517-6 (PMC11027328; doi:10.1186/s12871-024-02517-6)
Supplement: Supplementary file 2 — Supplementary Material 2 [file 12871_2024_2517_MOESM2_ESM.docx]

**Legend for Supplementary Video File**:

Video demonstration of the suggested technique for insertion of double lumen endotracheal tube. Length of video 2 minutes and 55 seconds.
